# Rapid loss of activity but not serum concentration in a passive infusion clinical trial of an HIV neutralizing antibody

**DOI:** 10.1101/2025.09.04.25334949

**Published:** 2025-09-07

**Authors:** Sharana Mahomed, Nonhlanhla N Mkhize, Leonid A. Serebryannyy, Tandile Hermanus, Prudence Kgagudi, Haajira Kaldine, Bronwen Lambson, Sandeep R. Narpala, Manjula Basappa, Mike Castro, Bob C Lin, Kevin Carlton, Jason T. Sands, Jason G. Gall, Richard A. Koup, Lynn Morris, Quarraisha Abdool Karim, Penny L. Moore, Salim Abdool Karim, Nicole Doria-Rose

**Affiliations:** 1. Centre for the AIDS Programme of Research in South Africa (CAPRISA), Durban, South Africa; 2. Department of Medical Microbiology, University of Kwazulu-Natal, Durban, South Africa; 3. SA MRC Antibody Immunity Research Unit, School of Pathology, Faculty of Health Sciences, University of the Witwatersrand, Parktown, Johannesburg 2193, South Africa; 4. Centre for HIV and STIs, National Institute for Communicable Diseases (NICD), a division of the National Health Laboratory Service, Johannesburg 2192, South Africa; 5. Vaccine Research Center, National Institute of Allergy and Infectious Diseases, National Institutes of Health, Bethesda, Maryland, USA; 6. Department of Public Health Medicine, School of Nursing and Public Health, University of KwaZulu-Natal, Durban, South Africa; 7. Institute of Infectious Disease and Molecular Medicine, University of Cape Town, Cape Town, South Africa; 8. Division of Medical Virology, Institute of Infectious Disease and Molecular Medicine, University of Cape Town, Cape Town, South Africa; 9. National Health Laboratory Services of South Africa; 10. Department of Epidemiology, Mailman School of Public Health, Columbia University, New York, USA

## Abstract

Monoclonal antibodies (mAbs) are a major class of drugs for treatment and prevention of disease. In early clinical trials, the pharmacokinetics (pK) of mAbs are usually assessed by measuring mAb concentration in serum. However, it is not a given that the mAbs will retain full functionality over time, emphasizing the need for integrated PK and functional assessments. In a re-analysis of data from the CAPRISA 012B trial, a previously published phase 1 study evaluating mAbs CAP256V2LS and VRC07-523LS in HIV-negative women, we report an unexpected disconnect between serum bNAb concentrations and HIV neutralization activity of CAP256V2LS, with implications for ongoing assessment of passive immunization trials.

HIV broadly neutralizing monoclonal antibodies (bNAbs) are being explored as long-acting HIV prevention tools, with multiple early-phase trials underway. The ability of bNAbs to prevent HIV infection has been demonstrated in numerous non-human primate studies, where bNAb levels correlated with protection.^2^ The AMP clinical trials showed that the VRC01 bNAb prevented acquisition of highly sensitive HIV strains.^3^ In published studies of several passively infused bNAbs in clinical trials, serum bNAb concentration and neutralization activity correlated closely over time, as expected.^4–7^

In CAPRISA 012B,^1^ we administered CAP256V2LS alone or with VRC07-523-LS in various regimens, comparing doses, co-administration of two antibodies, and route of delivery. This phase 1 trial was conducted at the CAPRISA eThekwini Clinical Research Site in Durban, South Africa. The protocol was reviewed and approved by the University of KwaZulu-Natal Biomedical Research Ethics Committee and the South African Health Products Regulatory Authority, and registered on the Pan African Clinical Trial Registry, PACTR202003767867253. Serum concentrations of each antibody were measured using anti-idiotype reagents specific to each bNAb, and neutralization titers (observed ID50) against strains that were sensitive to only one or the other antibody were measured with the TZM-bl Env-pseudovirus assay.

As previously reported,^1^ CAP256V2LS serum concentrations remained detectable for up to six months with an estimated half-life of 43 days, and no anti-drug antibody (ADA) was detected. However, comparison of the dynamics of neutralization vs serum concentration in serum showed a more rapid decline of neutralization titer than antibody concentration for CAP256V2LS, but not for VRC07-523LS. This is illustrated in Figure 1 for subjects receiving a 20 mg/kg single dose of each antibody co-administered. Predicted ID50 values were calculated as bNAb serum concentration divided by the IC50 of the tested virus. A faster decline in CAP256V2LS neutralization titers compared to serum concentration was reflected by the difference in predicted and observed ID50, and increased over time, indicative of loss of functional activity of the antibody present in the serum. In contrast, observed VRC07-523LS neutralization activity in the same participants tracked with the serum concentration, with the ratio between titer and concentration remaining consistent across all time points. The discordance between CAP256V2LS serum concentrations and neutralization activity was observed across all study arms including varying product dosages, routes of administration, presence or absence of recombinant human hyaluronidase (rHuPH20, product name Enhanze) used for subcutaneous administration, and single vs. co-bNAb administration (Supplementary Figures S1–S4).

This loss of *in vivo* functionality was specific to CAP256V2LS, even within donors who received both CAP256V2LS and VRC07-523LS. Potential mechanisms include immune complex formation, non-specific binding to serum proteins, loss of post-translational modifications such as tyrosine sulfation, and structural changes such as proteolytic cleavage, despite engineering to resist degradation.^8^ This phenomenon has not been observed in studies of other bNAbs, including VRC07-523LS (Figure 1) as well as 10E8VLS, N6LS, VRC01, and VRC01LS ^4–7^, which maintain proportional neutralization activity relative to serum concentrations. Defining the mechanism for this selective loss in neutralization of CAP256V2LS is the subject of ongoing studies, given that neutralization activity is a critical determinant of bNAb efficacy in preventing HIV^9^. Ongoing results from the CAPRISA 012C trial,^10^ expected in 2025, will clarify whether the observed decline in neutralization activity compromises its overall preventative efficacy. Investigators evaluating other monoclonal antibodies for infectious diseases and other conditions should remain vigilant for similar patterns and incorporate functional assays alongside assessments of serum antibody concentrations to better predict efficacy.

## Supplementary Material

1

## Figures and Tables

**Figure 1.**
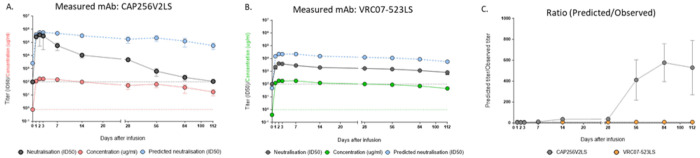
Comparison of bNAb concentration and neutralization activity in CAPRISA 012B participants. All subjects shown received a dual bNAb dose of 20 mg/kg body weight of CAP256V2LS (n=4) and VRC07-523LS (n=4), administered subcutaneously in the presence of Enhanze on Day 0. **A.** Concentration (μg/ml) (red line), measured neutralization titer (ID50) (black line) against HIV-CE2103_E8, and predicted neutralization (ID50) (concentration divided by the IC50, blue line) for CAP256V2LS. **B.** Concentration (μg/ml) (green line), measured neutralization titer (ID50) (black line) against HIV-Q769.D22, and predicted neutralization (ID50) (blue line) for VRC07-523-LS. **C.** Ratio of predicted ID50 to observed ID50 for CAP256V2LS (grey line) and VRC07-523-LS (orange line). Error bars show the standard deviation from the mean of participant data (n=4). The lower limit of detection (LOD) for the neutralisation assay is defined as an ID50 of 100. For PK assays the LOD was 0.8μg/ml and is shown by the red dotted lines
