# Supplementary material for: Rapid loss of activity but not serum concentration in a passive infusion clinical trial of an HIV neutralizing antibody
Source: medRxiv. 2025 Sep 7:2025.09.04.25334949. Preprint. [Version 1] doi: 10.1101/2025.09.04.25334949 (PMC12466785; doi:10.1101/2025.09.04.25334949)
Supplement: 1 [file NIHPP2025.09.04.25334949v1-supplement-1.pdf]

medRxiv preprint doi: https://doi.org/10.1101/2025.09.04.25334949; this version posted September 7, 2025. The copyright holder for this preprint (which was not certified by peer review) is the author/funder, who has granted medRxiv a license to display the preprint in perpetuity.
This article is a US Government work. It is not subject to copyright under 17 USC 105 and is also made available for use under a CC0 license.

## Supplementary Figures

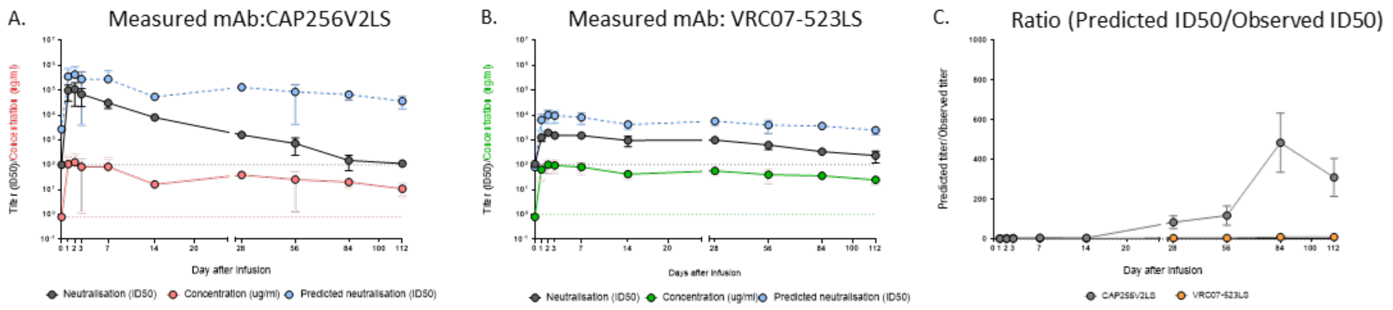


**Figure S1. Dual bNAb administration: 10mg/kg SC each, CAP256V2LS and VRC07-523LS.** Antibodies were administered together in the presence of Enhanze on Day 0. **A**. Concentration (µg/ml) (red line), measured neutralization titer (ID50) (black line) against HIV-CE2103_E8, and predicted neutralization (ID50) (concentration divided by the IC50, blue line) for CAP256V2LS. **B**. Concentration (µg/ml) (green line), measured neutralization titer (ID50) (black line) against HIV-Q769.D22, and predicted neutralization (ID50) (blue line) for VRC07-523LS. **C**. Ratio of predicted ID50 to observed ID50 for CAP256V2LS (grey line) and VRC07-523LS (orange line). Error bars show the standard deviation from the mean of participant data (n=4). The lower limit of detection (LOD) for the neutralisation assay is defined as an ID50 of 100. For PK assays the LOD was 0.8µg/ml and is shown by the red dotted lines

medRxiv preprint doi: https://doi.org/10.1101/2025.09.04.25334949; this version posted September 7, 2025. The copyright holder for this preprint (which was not certified by peer review) is the author/funder, who has granted medRxiv a license to display the preprint in perpetuity.
This article is a US Government work. It is not subject to copyright under 17 USC 105 and is also made available for use under a CC0 license.

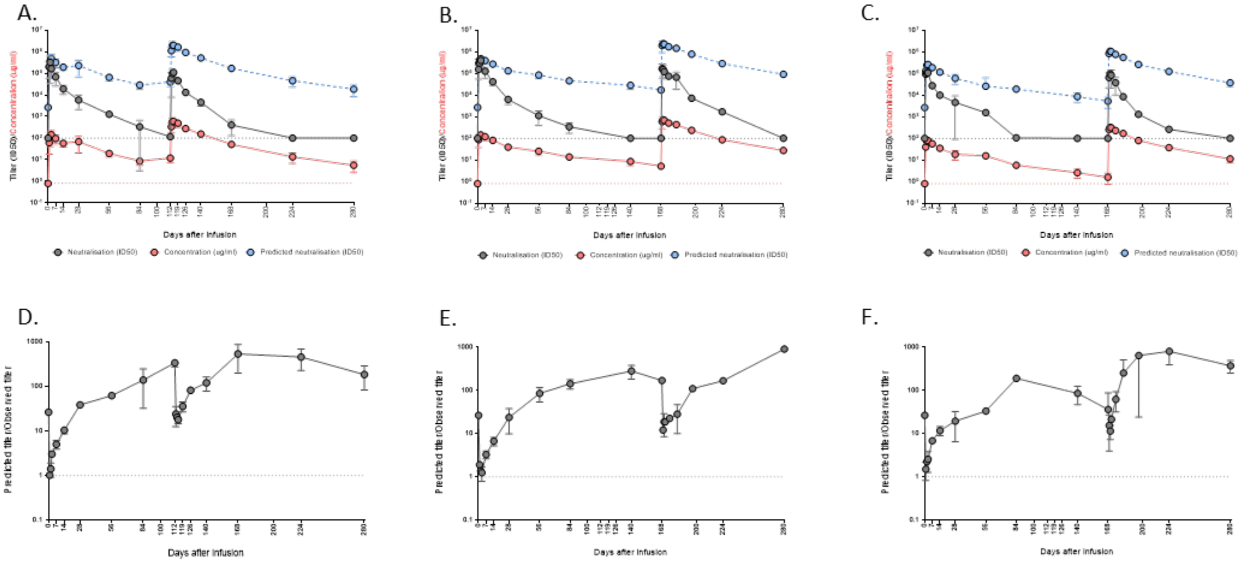


**Figure S2. Repeated doses of CAP256V2LS alone at Days 0 and 112 or Day 0 and 168.** Participants were given CAP256V2LS subcutaneously, in the presence of recombinant human hyaluronidase (rHuPH20), at doses of **A**. 10mg/kg, with repeated dose at Day 112 or **B.** 10mg/kg, with repeat dose at Day 168 or **C**. 20mg/kg, with repeat dose at Day 168. Red line: concentration (µg/ml). Black line: neutralization titer (ID50) against HIV-CE2103_E8. Blue line: predicted neutralization (ID50). The lower limit of detection (LOD) for the neutralisation assay is defined as an ID50 of 100. For PK assays the LOD was 0.8µg/ml and is shown by the red dotted lines. **D-F**. Ratios of predicted titer (ID50) to observed titer (ID50) for CAP256V2LS, corresponding to above panels. The black dotted line represents ratio (Predicted to Observed)=1. Error bars show the standard deviation from the mean of participant data (n=4).

medRxiv preprint doi: https://doi.org/10.1101/2025.09.04.25334949; this version posted September 7, 2025. The copyright holder for this preprint (which was not certified by peer review) is the author/funder, who has granted medRxiv a license to display the preprint in perpetuity.
This article is a US Government work. It is not subject to copyright under 17 USC 105 and is also made available for use under a CC0 license.

CAP256V2LS
5mg/kg IV

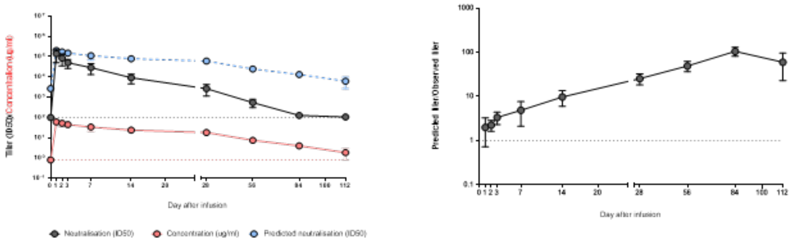

CAP256V2LS
10mg/kg IV

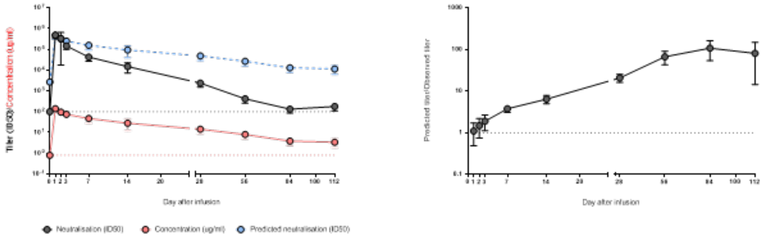

CAP256V2LS
5mg/kg SC

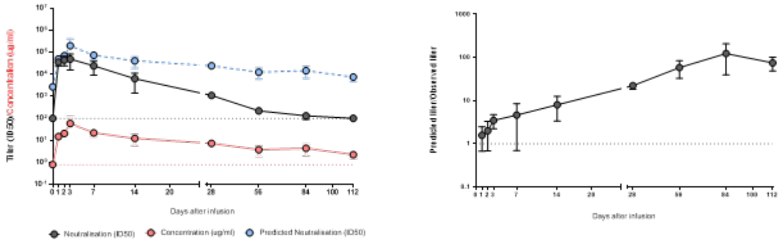

CAP256V2LS
5mg/kg SC*

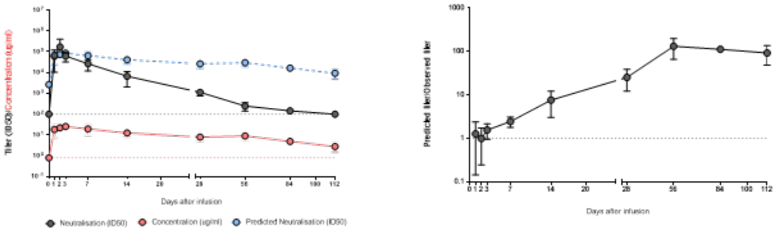

CAP256V2LS
10mg/kg SC*

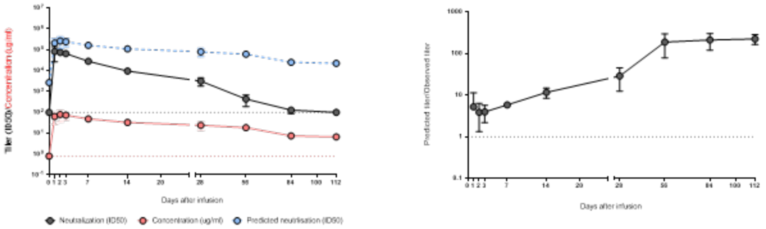

CAP256V2LS
20mg/kg SC*

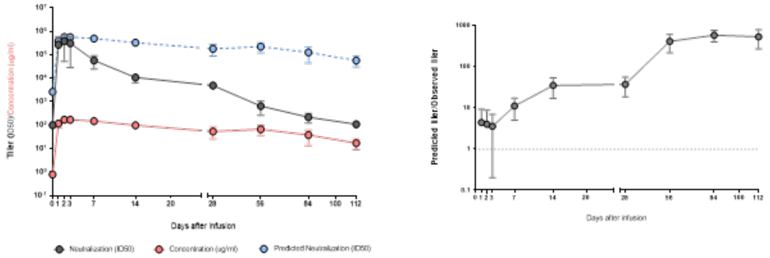

medRxiv preprint doi: https://doi.org/10.1101/2025.09.04.25334949; this version posted September 7, 2025. The copyright holder for this preprint (which was not certified by peer review) is the author/funder, who has granted medRxiv a license to display the preprint in perpetuity.
This article is a US Government work. It is not subject to copyright under 17 USC 105 and is also made available for use under a CC0 license.

**Figure S3. Single doses of CAP256V2LS alone.** 4 participants per group received a single dose of CAP256V2LS with the administration route and dosage amount shown. * indicates subcutaneous delivery in the presence of recombinant human hyaluronidase (rHuPH20). Left panels: Red line: concentration (µg/ml). Black line: measured neutralization titer (ID50) against HIV-CE2103_E8. Blue line: predicted neutralization (ID50) (concentration divided by the IC50). The lower limit of detection (LOD) for the neutralisation assay is defined as an ID50 of 100. For PK assays the LOD was 0.8µg/ml and is shown by the red dotted lines. Right panels: Ratios of predicted titer (ID50) to observed titer (ID50) for CAP256V2LS. The black dotted line represents ratio (Predicted to Observed)=1. Error bars show the standard deviation from the mean of participant data (n=4).

medRxiv preprint doi: https://doi.org/10.1101/2025.09.04.25334949; this version posted September 7, 2025. The copyright holder for this preprint
(which was not certified by peer review) is the author/funder, who has granted medRxiv a license to display the preprint in perpetuity.
This article is a US Government work. It is not subject to copyright under 17 USC 105 and is also made available
for use under a CC0 license.

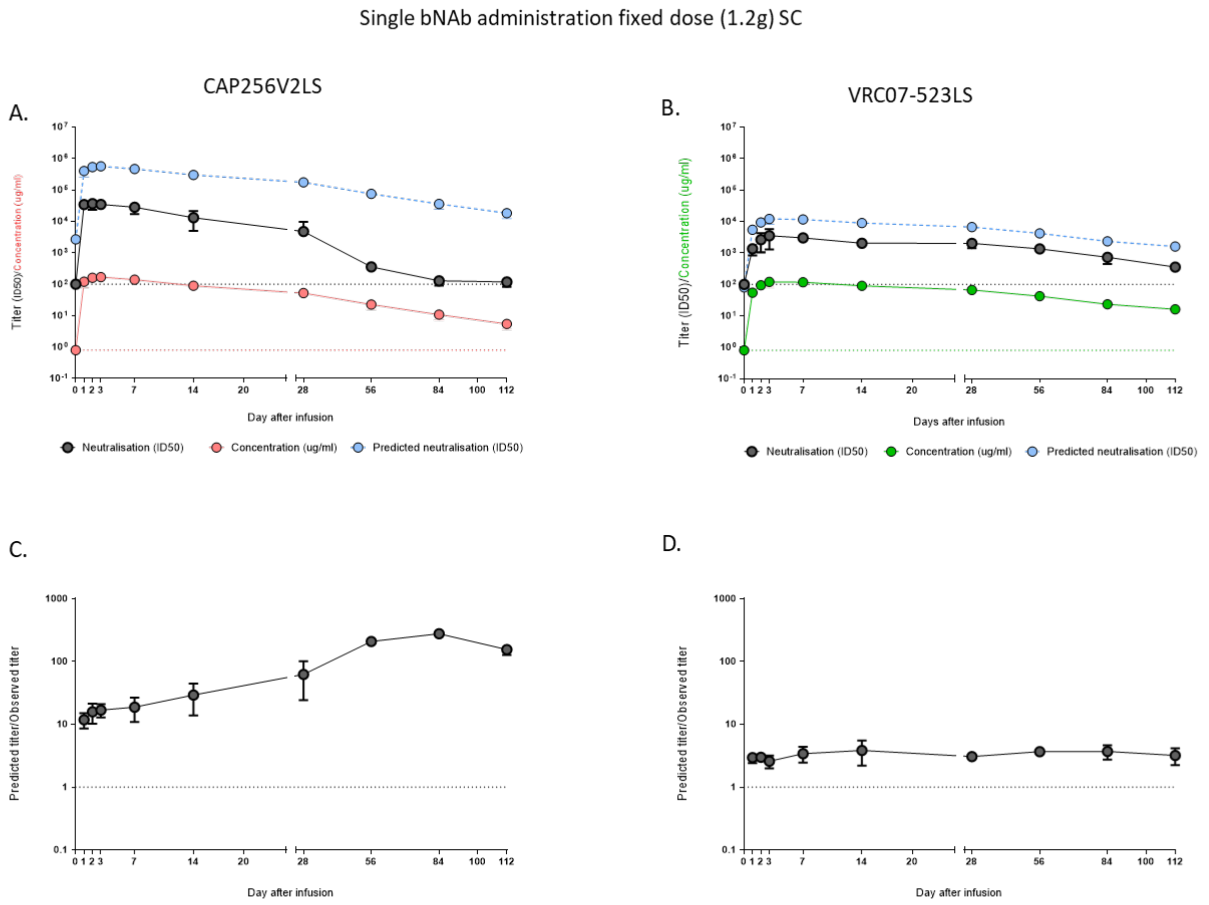


**Figure S4. Comparison of concentration and neutralization activity in CAP012B participants after administration of fixed dose CAP256V2LS or VRC07-523LS of 1.2 g.**

**A.** Lines show concentration (µg/ml) of CAP256V2LS (red line), measured neutralization titer (ID50) against HIV-CE2103 (black line), and predicted neutralization (ID50) (concentration divided by the IC50, blue line). The lower limit of detection (LOD) for the neutralization assay is defined as an ID50 of 100. For PK assays the LOD was 0.8µg/ml (red dotted lines). **B**. Lines show concentration (µg/ml) of VRC07-523-LS (red line), measured neutralization titer (ID50) against HIV-Q769.D422 (black line), and predicted neutralization (ID50) for VRC07-523LS. For PK assay, LOD was 1ug/ml (green dotted line), **C, D**. Ratio of predicted titer (ID50) to observed titer (ID50) for **C.** CAP256V2LS or **D**. VRC07-523-LS. The black dotted line represents ratio (Predicted to Observed) =1. Graphs display neutralization titers and concentrations from four participants per group. Error bars show the standard deviation from the mean of participant data (n=4)
